# Favorable Mortality-to-Incidence Ratio Trends of Lung Cancer in Countries with High Computed Tomography Density

**DOI:** 10.3390/medicina59020322

**Published:** 2023-02-09

**Authors:** Yao-Tung Wang, Brian-Shiian Chen, Han-Ru Wu, Ya-Chuan Chang, Chia-Ying Yu, Wen-Wei Sung

**Affiliations:** 1 Institute of Medicine, Chung Shan Medical University, Taichung 40201, Taiwan; 2 School of Medicine, Chung Shan Medical University, Taichung 40201, Taiwan; 3 Division of Pulmonary Medicine, Department of Internal Medicine, Chung Shan Medical University Hospital, Taichung 40201, Taiwan; 4 Department of Urology, Chung Shan Medical University Hospital, Taichung 40201, Taiwan

**Keywords:** lung cancer, mortality, incidence, mortality-to-incidence ratio, expenditure, computed tomography

## Abstract

*Background and Objectives*: The prognoses of lung cancer deteriorate dramatically as the cancer progresses through its stages. Therefore, early screening using techniques such as low-dose computed tomography (LDCT) is critical. However, the epidemiology of the association between the popularization of CT and the prognosis for lung cancer is not known. *Materials and Methods*: Data were obtained from GLOBOCAN and the health data and statistics of the World Health Organization. Mortality-to-incidence ratios (MIRs) and the changes in MIR over time (δMIR; calculated as the difference between MIRs in 2018 and 2012) were used to evaluate the correlation with CT density disparities via Spearman’s rank correlation coefficient. *Results*: Countries with zero CT density presented a relatively low incidence crude rate and a relatively high MIR in 2018 and a negative δMIR. Conversely, countries with a CT density over 30 had a positive δMIR. The CT density was significantly associated with the HDI score and MIR in 2018, whereas it demonstrated no association with MIR in 2012. The CT density and δMIR also showed a significant linear correlation. *Conclusions*: CT density was significantly associated with lung cancer MIR in 2018 and with δMIR, indicating favorable clinical outcomes in countries in which CT has become popularized.

## 1. Introduction

Lung cancer used to be a rare disease; however, since the beginning of the 21st century, it has become the cancer with the highest global incidence and mortality. In 2018, the number of new cases of lung cancer worldwide reached 2,093,876, and deaths due to lung cancer totaled 1,761,007 [1]. These data are striking and have aroused public concern. Moreover, mortality rates in 2018 closely paralleled the incidence rate of lung cancer worldwide, meaning that treatment outcomes are poor for lung cancer patients after diagnosis [1]. Lung cancer is usually diagnosed in advanced stages [2], and the prognosis during these stages is extremely poor. Early diagnosis is clearly a matter of public interest.

When lung cancer is diagnosed at an early stage, the five-year survival rate can rise to 60–80%, much higher than after diagnoses at stage 3 (16%) and stage 4 (less than 10%) [3]. Unfortunately, early diagnosis is quite difficult since lung cancer in its early stage is asymptomatic, and its detection depends on radiographic imaging, such as X-ray or computed tomography (CT). However, compared with CT screening, the use of X-rays to find abnormalities in lung cancer, especially in small lesions, is far more challenging. In 90% of early lung cancer cases, misdiagnoses are frequent when X-rays are used [4], mainly due to the difficulty in distinguishing lung lesions from bones, pulmonary vessels, mediastinal structures, and other complex anatomical structures [4]. Conversely, CT images are more advantageous because they provide a series of cross-sectional images of the pulmonary regions, which can help medical practitioners distinguish 83% to 91% of these lesions [5].

Low-dose computed tomography (LDCT), as the name suggests, allows screening at a lower radiation dose (1.0–1.4 mSV) than is conventionally used with CT [6]. Moreover, related research has shown no significant differences between LDCT and CT when used for lung cancer screening, as the concordance rate for diagnoses is approximately 80% [7]. CT scanners are mainly used for the detection of suspicious lung nodules and the establishment of baseline screening for lung cancer [8]. However, the density of CT scanners, defined as the total number of CT facilities per million people, varies dramatically from region to region across the globe. The expenses for the acquisition and maintenance of CT facilities can be enormous; consequently, the construction of these high-end medical devices can be influenced by the level of economic development of a given country. Given the important role that LDCT plays in lung cancer prognosis and the regional differences in the density of CT scanners, we proposed that CT density might affect the worldwide mortality-to-incidence ratios (MIRs) for lung cancer. Many previous studies have focused on the effectiveness of LDCT by analyzing the correlation between regular LDCT screening and lung cancer mortality rates [5,9], whereas few studies have explored the real situation regarding the availability of LDCT worldwide. For this reason, we have sought to provide a comprehensive view of the relevance of CT availability to lung cancer prognosis. By analyzing global epidemiological data, our aim in conducting this study is to determine the association between the CT density and MIRs.

## 2. Materials and Methods

The disease code for the study is based on the International Classification of Diseases, 10th Revision, Clinical Modification (ICD-10-CM). Individuals with the ICD-10-CM code (ICD-10-CM C33-34) are regarded as having been diagnosed with lung cancer. Epidemiological data for 185 regions between 2012 and 2018 were obtained from the GLOBOCAN database (https://gco.iarc.fr/today/ (accessed on 26 September 2020)). The human development index (HDI) was obtained from the United Nations Development Programme, Human Development Report Office (http://hdr.undp.org/en (accessed on 26 September 2020)). Data for the density of CT facilities for 2013 were obtained from the Global Health Observatory data repository (https://www.who.int/data/gho (accessed on 26 September 2020)) and were defined as the number of CT units per million people. The MIR was defined as the ratio of the crude rate of mortality to the crude rate of incidence, as described in the previous literature [10,11,12,13]. The δMIR was defined as the difference between the MIR values of 2012 and those of 2018 (δMIR = MIR [in 2012] − MIR [in 2018]) [14].

The exclusion criteria for country selection included missing data for the density of CT facilities (N = 60), missing data for MIR/HDI (N = 3), and outliers for the density of CT facilities (N = 2). A total of 115 countries were considered eligible for the final analysis.

The associations between the MIR, δMIR, and other factors among various countries were estimated and gauged using Spearman’s rank correlation coefficient with the SPSS statistical software version 15.0 (IBM, Inc., Chicago, IL, USA). Values of *p* < 0.05 were defined as statistically significant. Scatterplots were generated using SigmaPlot software (Systat Software Inc., San Jose, CA, USA).

## 3. Results

### 3.1. Human Development Index and the CT Density in Selected Countries

Table 1 shows the scores and ranks for the human development index and the CT density in each country. The results are listed in alphabetical order by country name. Nations whose CT densities are less than 0.01 are the Central African Republic, Guinea-Bissau, Guinea, and Vanuatu. As expected, these countries also rank low in terms of HDI (187, 174, 176, and 122, respectively). By contrast, the countries with a CT density higher than 20 all rank in the top 50 for the HDI score, except for Lebanon, which ranks 70th. The country with the highest CT density is Iceland (39.45), while countries with the lowest CT densities are the Central African Republic, Guinea-Bissau, Guinea, and Vanuatu (0.00), whose HDI scores rank 10, 172, 161, 162, and 122, respectively.

### 3.2. CT Density and Incidence Crude Rates, MIR, and MIR Disparity in Lung Cancer

Countries without any CT scanners are the Central African Republic, Guinea-Bissau, Guinea, and Vanuatu, all of which have crude incidence rates lower than 10 (2.2, 2.8, 2.4, and 8.0, respectively). Furthermore, their MIRs in 2018 were all higher than 0.9, and the values of δMIR were all negative. In terms of δMIR, the countries whose CT densities were all above 30, such as Iceland, Greece, and South Korea, presented positive values for δMIR (0.15, 0.12, and 0.11, respectively). Conversely, Vanuatu had an extremely low δMIR (−0.14) and the highest MIR in 2018 (1.06), with a CT density of zero. The mean and standard deviation (S.D.) of MIRs in 2012 and 2018 were 0.90 ± 0.07 and 0.91 ± 0.09, respectively. Their paired samples correlation was −0.189 (*p* = 0.043), and the difference was statistically significant (in the paired *t*-test, 95% confidence interval: −0.240 to −0.172, *p* < 0.001).

### 3.3. CT Density Is Significantly Associated with the HDI Score

**Figure 1:**
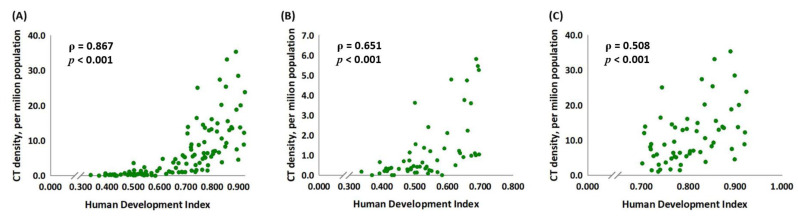
The human development index of (**A**) all selected countries (*n* = 115) and of countries with (**B**) HDI scores <0.70 (*n* = 56) and (**C**) HDI scores ≥0.70 (*n* = 59) are significantly associated with CT density.

Figure 1 presents the association between the CT density and the HDI score according to all selected countries, grouped according to their HDI scores (<0.70 and ≥0.70, respectively). All three groups showed a significant association between the CT density and the HDI (*ρ* = 0.867, *p* < 0.001, Figure 1A; *ρ* = 0.651, *p* < 0.001, Figure 1B; *ρ* = 0.508, *p* < 0.001, Figure 1C).

### 3.4. The Association between CT Density and MIR in 2012 and 2018 and δMIR

**Figure 2:**
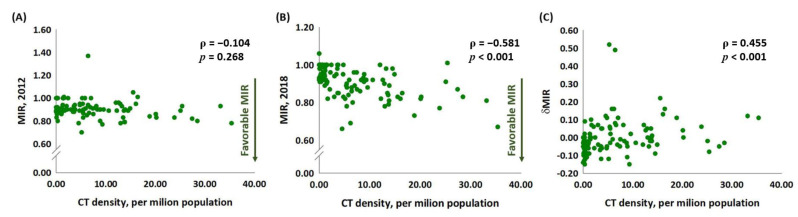
Lack of an association between the CT density and the MIR in 2012 (**A**). However, CT density was significantly associated with (**B**) MIR in 2018 and (**C**) δMIR in lung cancer.

No association was noted between the CT density and the MIR in 2012 (ρ = −0.104, *p* = 0.268, Figure 2A), whereas the CT density was significantly associated with the MIR in 2018, as shown in Figure 2 (ρ = −0.581, *p* < 0.001, Figure 2B). The linear correlation between the CT density and δMIR revealed a significant association (ρ = 0.455, *p* < 0.001, Figure 2C). We compared the MIRs and δMIR according to the CT densities divided into three groups (CT density <1 as a reference, compared with CT density between 1 and <10 and CT density ≥10). The MIR in 2012 showed no statistically significant difference between the three groups (MIR [mean ± S.D.]: 0.91 ± 0.05, 0.91 ± 0.09, and 0.88 ± 0.07 for CT density <1, 1–10, and ≥10, respectively; *p* = 0.586 and *p* = 0.160 for CT density 1–10 and ≥10 compared with CT density <1, respectively). For the MIR in 2018, the countries with CT density 1–10 and ≥10 had significantly lower MIRs (MIR [mean ± S.D.]: 0.97 ± 0.04, 0.89 ± 0.09, and 0.85 ± 0.09 for CT density <1, 1–10, and ≥10, respectively; *p* < 0.001 and *p* < 0.001 for CT density 1–10 and ≥10 compared with CT density <1, respectively). The countries with CT density 1–10 and ≥10 had significantly favorable δMIR (δMIR [mean ± S.D.]: −0.06 ± 0.05, 0.02 ± 0.12, and 0.03 ± 0.08 for CT density <1, 1–10, and ≥10, respectively; *p* < 0.001 and *p* < 0.001 for CT density 1–10 and ≥10 compared with CT density <1, respectively). These results showed that the CT density in 2013 did not significantly change the MIR in 2012 but caused a statistically significant change in the MIR in 2018 and in the δMIR.

## 4. Discussion

This study evaluated the correlation between the human development index and the CT density per million people. The use of data collected from GLOBCAN and WHO World Health Statistics revealed a strong association between the CT density and the HDI score. We conducted a further series of subgroup analyses on the countries with HDI scores of <0.70 and ≥0.70, and the results also demonstrated similar trends. Next, we assessed the association between mortality and the incidence rate of lung cancer versus the CT density. The association between the MIR of lung cancer and the CT density was negative in 2018, but no statistically significant association was observed for the data collected in 2012. However, the analysis of the CT density and the difference between the MIRs in 2012 and 2018 revealed a positive correlation between the CT density and δMIR. Our findings suggest a positive intercorrelation between socioeconomic status and the CT density, which may serve as an indicator of medical investment. In addition to this intercorrelation, our study revealed a gradual improvement in the quality of medical care globally.

Among all cancer types, lung cancer is the type that is most commonly diagnosed, and it was the globally leading cause of death for men and the third leading cause for women in 2018 [1]. Although many risk factors are responsible for the carcinogenesis of lung cancer (e.g., occupational exposures to asbestos-related substances, air pollution, and smoke from pristine coal [15,16,17]), approximately 90% of lung cancer cases are attributed to tobacco smoking [18]. Nicotine addiction has always been a grave public health issue because of the health hazards smokers experience after long-term cigarette use, especially the elevated risk of developing lung cancer. The process of burning involved in smoking generates countless carcinogens (e.g., polycyclic aromatic hydrocarbon and N-nitrosamines) as well as a variety of oxidants in both the tar and the gas (i.e., free radicals and reactive oxygen species) [19,20]. The mechanistic effects and roles played by these substances in the carcinogenesis of lung cancer have been identified. The compounds generated by burning cigarettes may disrupt normal genetic functions by forming covalent bonds with DNA, inducing transversions of the nucleotides, and harming intracellular structures. The end result can be the activation of oncogenes, the inactivation of tumor-suppressor genes, and ultimately the formation of pulmonary tumors [19,21,22].

The diagnosis and staging of lung cancer lesions entail radiographic imaging. Tumors usually appear in the form of white bulks in conventional X-ray screening, and ambiguity in the images may complicate the differentiation of potential neoplasms from other tissues, organs, or pulmonary symptoms. Currently, CT is the more preferable and most widely adopted approach to lung cancer imaging for almost all purposes [23]. CT with a lower dose of radiation, termed LDCT, is commonly used in procedures involved in diagnosing pulmonary neoplasms [6]. CT images provide a better resolution of imaging for many reasons. Unlike conventional radiography, the X-ray tubes in a CT scanner that project X-ray beams travel in a round-shaped gantry. Because they revolve around the patient while the patient is sent through the gantry, the detectors on the opposite side of the X-ray tube receive the signals that have penetrated and convert them into cross-sectional images that precisely capture the pulmonary structures. These two-dimensional pictures can be integrated into three-dimensional versions, enabling medical practitioners to observe the structures of skeptical masses or nodules with sufficient spatial information [24].

The use of positron emission tomography in conjunction with CT is another derivative of CT and is also an advanced approach for the detection of lung cancer. Following the injection of radioactive tracers, the compounds may aggregate in lesions, as the lesions typically display metabolic abnormalities. With the aid of PET and CT scanners, the exact site of the tumor can be pinpointed in quantitative images [25].

Numerous studies have examined the benefits of LDCT screening. The large National Lung Screening Trial (NLST), with an enrollment of 53,454 persons, focused on the effect of routine LDCT screening on mortality reduction but only concluded that ever-smokers were at high risk [26,27]. Our study, which is also based on the NLST, generally explored the association between CT density distribution and the prognosis of lung cancer and revealed a concordance between the geographical distribution of actual CT density and the prognosis of lung cancer. Adequate CT facilities might better meet the needs of people eligible for routine screening, thereby improving the prognosis of lung cancer.

According to the NLST, LDCT can reduce lung cancer mortality by 20% compared with X-rays, and follow-up European studies also support the effectiveness of LDCT [28,29]. LDCT mainly detects nodules in the lungs. The size, growth rate, morphology, and location of the nodules are references used for malignant or benign judgments. Related research shows that most lung cancer is confirmed in large nodules, whereas lung nodule counts have yet to be confirmed to determine malignancy [30]. Compared with the high misdiagnosis rate and the increased cost and time-consuming shortcomings of MIRs, regular LDCT screening might be a more practicable form of radiography for the close tracking of new large nodules [4,31]. Due to the effectiveness of LDCT screening, the United States now recommends that current and former smokers aged 55 to 80 years with a 30-year history of smoking receive routine LDCT screening [32]. The recommendation for LDCT screening of high-risk people was based on NLST research and was announced in 2011 [26,32]. This might explain why we failed to identify a significant association between CT density and MIR in the analysis we conducted in 2012 and the subsequent significant association we found in our analysis conducted in 2018.

The CT density is also significantly associated with HDI, which is a proxy for the degree of socioeconomic development. Three major factors are included in the HDI estimation: years of education received by people aged above 25 or expected to be received for those below 25, gross national income per capita (GNI), and life expectancy [33]. Thus, the HDI score may serve as a relatively objective indicator of the overall performance of a particular society. According to previous research, CT has an incremental cost-effectiveness ratio (ICER) of GBP 10,069 per quality-adjusted life year (QALY) [34]. This number might represent an imposing burden on low-HDI countries in which primary healthcare systems remain to be developed. In other words, our findings suggest that CT density might also reflect the amount of money invested in medical care. A low CT density could also correlate with lower quality and standards of care for lung cancer patients. The procedures for diagnosis could be inaccurate and unthorough, drawing insufficient data for accurate evaluations. For cases discovered by qualified medical partitioners, a lack of access to the standard and optimal regimens, such as surgery, chemotherapy, radiotherapy, immunotherapy, and target therapy, in those places may lead to a worse prognosis of lung cancer. Therefore, a substantial number of cases could potentially remain either unreported or untreated in countries that have few CT scanners, suggesting a worse and unequal status for lung cancer patients in places that lack proper healthcare systems.

This study has some limitations. First, the study was chiefly based on second-hand information. Any mistakes made in the first place could not be known, but they could certainly influence the outcome of our analysis. Second, only the countries with a CT density recorded by the WHO were included in our study. Approximately 70 nations, ranging from well-developed to under-developed countries, were not incorporated in this research due to missing data. The incompleteness of all the general information could have resulted in a failure to genuinely reflect the real-world situations. Third, the level of CT density might not be representative of the people who received routine screening. Other clinical objectives, such as the diagnosis of neurological, gastrointestinal, and cardiological diseases, are also reliant on CT screening. Hence, the deployment of CT scanners may be representative of more than just screening for pulmonary tumors. Moreover, histopathological examination is a relatively definitive process for determining lung cancer lesions. Therefore, the CT density may only indirectly manifest the prognostic outcomes, rather than being an absolute index. The fourth limitation of the study is that previous research has not shown any significant association between LDCT and a decrease in lung cancer mortality rates in males, meaning that the study might have ignored sex differences [5]. Last, many factors can affect the outcome of MIR. The baseline physical profiles of the patients and the stages at which the lesions were diagnosed may have an impact on the mortality rate, which may vary from place to place [35,36]. Each subtype of lung cancer also possesses unique pathological traits. For instance, the prognosis of small cell lung cancer accounts for 15% of all lung cancer cases, but this small cell type of lung cancer appears to be more aggressive than non-small cell lung cancer, which accounts for about 85% of cases. The quality of care—and the accessibility of this care—following the initial diagnosis may also have a substantial impact on the mortality rate of lung cancer. Inequality may also appear in different countries in terms of lung cancer treatments, including surgery (i.e., lobectomy and pneumonectomy), chemotherapy, immunotherapy, and radiotherapy. Unfortunately, not all the clinical information was available. Consequently, further investigation and adjustment for these variables were not possible in our study. Additional studies are required in the future to determine whether these variables are potential confounders that affect the results. Despite these limitations, our findings still demonstrate that the prognosis of lung cancer might improve as the CT density rises. To the best of our knowledge, our study is the first to identify an association between the CT density and either the HDI scores of countries or the MIRs of lung cancer using recent global data. Collectively, our discoveries should provide new insights into HDI, lung cancer, and the global distribution of CT scanners in public health.

## 5. Conclusions

The CT density was significantly associated with the MIR in 2018 and with the MIR trend, δMIR, indicating a favorable prognosis for lung cancer patients in countries in which CT has become popularized.

## Figures and Tables

**Table 1:** Summary of human development index, CT density, cancer incidence, cancer mortality, and mortality-to-incidence ratio for lung cancer in selected countries.

	Human Development Index			Incidence			Mortality			Mortality-to-Incidence Ratio		
Country	Score	Rank	CT Density	Number	ASR	CR	Number	ASR	CR	2012	2018	δMIR
Afghanistan	0.479	153	0.20	1019	2.8	6.1	1022	2.8	6.1	0.87	1.00	−0.13
Albania	0.771	61	5.36	1087	37.4	21.1	961	33.1	18.1	0.95	0.89	0.06
Angola	0.537	135	0.42	401	1.3	3.2	396	1.3	3.2	0.88	1.00	−0.12
Armenia	0.737	75	3.02	1266	43.7	27.8	1186	40.9	26.1	0.90	0.94	−0.04
Austria	0.899	15	28.49	4845	56.9	27.3	4012	47.1	21.1	0.80	0.83	−0.03
Azerbaijan	0.736	78	1.06	1346	13.6	12.5	1265	12.8	11.7	0.88	0.94	−0.06
Bahamas	0.797	51	13.25	33	8.3	6.1	32	8.1	6.0	0.93	0.98	−0.05
Barbados	0.811	44	7.03	46	16.3	8.7	41	14.5	7.6	1.00	0.89	0.11
Belarus	0.803	46	6.20	4118	44.3	25.0	2839	30.5	17.0	0.85	0.69	0.16
Belize	0.706	90	12.05	25	6.6	9.8	25	6.6	9.8	0.96	1.00	−0.04
Benin	0.489	149	0.29	65	0.6	1.0	64	0.6	1.0	1.00	0.98	0.02
Bhutan	0.591	120	1.33	48	5.9	7.9	44	5.4	7.3	0.94	0.92	0.02
Bosnia andHerzegovina	0.741	73	16.45	2379	69.1	35.9	2034	59.0	29.5	1.01	0.85	0.16
Botswana	0.687	96	0.99	45	1.9	2.9	44	1.9	2.9	0.91	1.00	−0.09
Burkina Faso	0.394	170	0.65	243	1.2	2.7	231	1.2	2.6	0.89	1.00	−0.11
Burundi	0.416	166	0.20	86	0.8	1.6	81	0.7	1.6	0.89	0.94	−0.05
Cambodia	0.548	129	1.19	1544	9.5	13.0	1497	9.2	12.6	0.88	0.97	−0.09
Cameroon	0.524	138	0.63	294	1.2	2.3	293	1.2	2.3	0.89	1.00	−0.11
Canada	0.906	12	13.76	22,340	61.9	28.4	17,566	48.7	21.7	0.79	0.79	0.00
Central African Republic	0.370	172	0.00	56	1.2	2.2	54	1.1	2.1	0.89	0.92	−0.03
Chad	0.393	171	0.08	102	0.7	1.5	95	0.6	1.5	0.83	0.93	−0.10
Chile	0.818	43	12.60	3432	19.1	12.5	3163	17.6	11.5	0.96	0.92	0.04
Comoros	0.529	137	1.36	1	0.1	0.2	1	0.1	0.2	1.00	1.00	0.00
Costa Rica	0.774	59	5.13	405	8.3	6.1	335	6.8	4.9	0.70	0.82	−0.12
Côte d’Ivoire	0.467	155	0.69	276	1.1	2.2	267	1.1	2.2	0.90	1.00	−0.10
Croatia	0.820	42	14.92	2817	69.3	31.7	2684	66.0	29.7	0.91	0.95	−0.04
Cuba	0.764	66	4.79	6318	56.1	29.9	5267	46.8	24.5	0.94	0.83	0.11
Cyprus	0.852	30	25.41	454	38.7	23.0	456	38.9	22.7	0.93	1.01	−0.08
Czechia	0.865	26	12.99	6204	59.6	26.5	4821	46.3	20.2	0.78	0.78	0.00
Denmark	0.924	5	23.85	4546	80.8	35.2	3487	62.0	25.5	0.83	0.77	0.06
Dominican Republic	0.708	89	13.89	1236	11.5	11.7	1106	10.2	10.3	0.90	0.89	0.01
Ecuador	0.740	74	1.59	965	5.8	5.5	888	5.3	5.0	1.01	0.91	0.10
El Salvador	0.660	107	4.73	362	5.7	4.9	343	5.4	4.6	0.89	0.95	−0.06
Eritrea	0.422	165	0.32	79	1.5	2.8	77	1.5	2.8	0.91	1.00	−0.09
Estonia	0.859	27	15.54	790	62.1	28.8	653	51.3	22.7	1.05	0.83	0.22
Eswatini/Swaziland	0.542	133	2.40	18	1.3	2.5	18	1.3	2.6	1.00	1.00	0.00
Ethiopia	0.429	163	0.36	2033	1.9	3.5	2032	1.9	3.5	0.89	1.00	−0.11
Fiji	0.702	91	3.40	54	5.9	5.9	51	5.6	5.6	0.88	0.95	−0.07
Finland	0.908	9	20.09	2480	46.0	18.4	2035	37.7	14.7	0.86	0.82	0.04
Gabon	0.672	103	3.59	95	4.6	6.7	92	4.5	6.6	0.93	0.98	−0.05
Georgia	0.749	69	8.75	1148	29.8	16.7	1070	27.8	15.9	0.90	0.93	−0.03
Ghana	0.570	124	0.15	234	0.8	1.4	217	0.7	1.3	0.86	0.93	−0.06
Greece	0.856	28	33.16	9229	85.6	39.6	7498	69.5	30.8	0.93	0.81	0.12
Guinea-Bissau	0.437	161	0.00	18	0.9	1.8	18	0.9	1.8	1.00	1.00	0.00
Guinea	0.431	162	0.00	184	1.4	2.4	165	1.3	2.2	0.88	0.93	−0.05
Guyana	0.652	108	3.75	21	2.7	3.0	21	2.7	3.0	0.94	1.00	−0.06
Haiti	0.478	154	0.29	491	4.4	5.9	441	4.0	5.4	0.91	0.91	0.00
Honduras	0.600	118	2.10	363	3.9	5.5	321	3.4	4.8	0.93	0.87	0.06
Hungary	0.826	39	6.63	10,550	111.1	55.8	8457	89.0	43.5	0.87	0.80	0.07
Iceland	0.908	10	39.45	174	52.5	29.6	125	37.7	19.4	0.87	0.72	0.15
Iraq	0.662	106	2.22	2075	5.3	10.4	2019	5.1	10.1	0.90	0.96	−0.06
Ireland	0.899	16	4.54	2694	56.9	31.8	1778	37.6	20.2	0.78	0.66	0.12
Israel	0.893	19	7.50	2304	27.7	20.1	1997	24.0	17.1	0.86	0.87	−0.01
Jamaica	0.722	84	1.44	481	16.8	13.2	439	15.3	11.9	0.90	0.91	−0.01
Jordan	0.726	83	5.50	1093	11.1	17.3	972	9.8	15.6	0.90	0.88	0.02
Kazakhstan	0.782	57	1.46	4239	23.1	21.3	3798	20.7	19.1	0.90	0.90	0.00
Kenya	0.545	132	0.25	665	1.3	2.9	651	1.3	2.9	0.92	1.00	−0.08
Kyrgyzstan	0.649	109	0.90	657	10.7	13.9	606	9.9	12.7	0.89	0.93	−0.04
Laos	0.569	125	0.74	861	12.4	18.3	829	11.9	17.8	0.87	0.96	−0.09
Lebanon	0.744	70	25.09	1546	25.5	22.2	1396	23.1	19.9	0.89	0.91	−0.02
Lithuania	0.835	36	20.22	1530	54.6	26.0	1269	45.3	21.1	0.83	0.83	0.00
Luxembourg	0.892	20	18.85	278	48.1	27.0	203	35.1	19.1	0.84	0.73	0.11
Madagascar	0.507	141	0.13	148	0.6	1.0	133	0.5	0.9	0.89	0.91	−0.02
Malawi	0.452	158	0.31	127	0.7	1.5	121	0.6	1.4	0.80	0.95	−0.15
Malaysia	0.782	56	6.43	4547	14.2	14.7	3903	12.2	12.6	0.94	0.86	0.08
Maldives	0.688	95	5.80	38	8.6	11.9	32	7.2	10.3	1.00	0.84	0.16
Mali	0.408	168	0.20	246	1.3	3.0	239	1.3	3.0	0.91	1.00	−0.09
Malta	0.854	29	9.32	184	43.3	18.3	170	40.0	16.4	0.77	0.92	−0.15
Mauritania	0.503	143	1.54	60	1.3	2.4	60	1.3	2.4	1.00	1.00	0.00
Mauritius	0.768	63	6.43	196	15.6	9.9	172	13.7	8.6	1.37	0.88	0.49
Mexico	0.752	68	3.65	6952	5.4	5.4	5921	4.6	4.6	0.90	0.85	0.05
Moldova	0.693	94	5.45	1678	41.9	27.3	1302	32.5	21.0	0.83	0.78	0.05
Mongolia	0.719	87	8.10	429	13.8	18.6	368	11.8	16.1	0.93	0.86	0.07
Montenegro	0.798	49	16.09	407	65.6	39.2	333	53.6	30.6	0.95	0.82	0.13
Morocco	0.636	111	1.21	6391	17.7	17.0	6303	17.5	16.8	0.90	0.99	−0.09
Myanmar	0.541	134	0.08	7524	14.0	14.8	7347	13.7	14.5	0.89	0.98	−0.09
Namibia	0.612	116	4.78	62	2.4	4.2	61	2.4	4.1	0.95	1.00	−0.05
Netherlands	0.921	6	12.23	11,713	70.1	32.4	9652	57.8	24.9	0.89	0.82	0.07
Nicaragua	0.625	114	0.49	289	4.6	5.5	269	4.3	5.1	0.90	0.93	−0.03
Niger	0.338	173	0.17	40	0.2	0.4	40	0.2	0.4	1.00	1.00	0.00
Oman	0.804	45	6.88	109	2.3	4.5	106	2.2	4.4	0.92	0.96	−0.04
Pakistan	0.533	136	0.33	9574	4.8	6.9	9069	4.5	6.6	0.87	0.94	−0.07
Panama	0.770	62	9.58	378	9.2	8.2	333	8.1	7.2	0.90	0.88	0.02
Papua New Guinea	0.508	140	0.41	603	7.2	11.6	597	7.1	11.5	0.88	0.99	−0.11
Paraguay	0.697	92	1.03	700	10.2	11.2	667	9.7	10.7	0.89	0.95	−0.06
Philippines	0.684	98	1.09	16,597	15.6	19.9	14,803	13.9	17.9	0.86	0.89	−0.03
Poland	0.836	35	10.60	26,968	72.3	35.7	24,910	66.7	32.3	0.89	0.92	−0.03
Portugal	0.829	38	27.43	4766	47.8	21.9	4144	41.5	18.2	0.82	0.87	−0.05
Qatar	0.850	31	8.30	71	2.6	7.8	66	2.4	7.5	0.90	0.92	−0.02
Romania	0.796	52	5.44	10,862	56.6	29.3	9804	51.1	25.9	0.87	0.90	−0.03
Samoa	0.696	93	5.25	56	28.5	34.2	27	13.7	16.3	1.00	0.48	0.52
Saudi Arabia	0.837	34	3.82	898	2.7	4.1	754	2.3	3.6	0.90	0.85	0.05
Senegal	0.489	148	0.35	182	1.1	2.2	170	1.0	2.1	0.89	0.91	−0.02
Serbia	0.772	60	13.67	7851	91.1	49.8	6619	76.8	39.4	0.89	0.84	0.05
Sierra Leone	0.413	167	0.33	84	1.1	2.3	80	1.0	2.2	1.00	0.91	0.09
Slovenia	0.876	23	13.51	1390	68.5	32.1	1176	58.0	26.1	0.83	0.85	−0.02
South Africa	0.673	102	0.97	7867	13.7	16.4	7398	12.9	15.5	0.90	0.94	−0.04
South Korea	0.890	21	35.38	26,285	52.1	26.0	17,579	34.8	16.2	0.78	0.67	0.11
Spain	0.873	25	13.85	24,812	55.3	26.3	19,998	44.6	20.4	0.79	0.81	−0.02
Sri Lanka	0.762	67	1.69	1386	6.7	5.0	1143	5.5	4.1	0.89	0.82	0.07
Sudan	0.485	150	1.13	541	1.3	2.2	508	1.2	2.1	0.90	0.92	−0.02
Suriname	0.720	86	7.42	95	16.8	15.4	91	16.1	14.8	0.91	0.96	−0.05
Tajikistan	0.639	110	1.10	322	3.5	5.4	304	3.3	5.1	0.91	0.94	−0.03
Tanzania	0.501	145	0.12	149	0.3	0.5	148	0.3	0.5	1.00	1.00	0.00
Thailand	0.733	81	5.95	21,492	31.4	18.9	19,816	29.0	17.8	0.91	0.92	−0.01
Togo	0.484	151	0.73	75	0.9	1.9	74	0.9	1.8	0.88	0.99	−0.11
Trinidad andTobago	0.784	55	2.98	239	17.5	12.4	197	14.5	10.2	0.90	0.83	0.07
Tunisia	0.721	85	8.91	1851	16.0	13.6	1760	15.2	13.0	0.90	0.95	−0.05
Turkey	0.765	65	14.52	33,235	40.8	35.6	32,377	39.8	34.8	0.89	0.98	−0.09
Uganda	0.497	147	0.45	464	1.0	2.7	439	1.0	2.7	0.91	0.99	−0.08
Uruguay	0.788	53	12.91	1452	42.9	27.2	1315	38.8	24.2	0.95	0.90	0.05
Vanuatu	0.584	122	0.00	14	5.0	8.0	15	5.3	8.7	0.92	1.06	−0.14
Yemen	0.501	146	3.61	544	1.9	3.9	542	1.9	3.9	0.88	1.00	−0.12
Zambia	0.552	128	0.21	233	1.3	3.4	228	1.3	3.4	1.00	1.00	0.00
Zimbabwe	0.516	139	0.42	310	1.8	4.0	298	1.8	4.0	0.90	1.00	−0.10

## Data Availability

The datasets used and/or analyzed during the current study are publicly available in the Global Cancer Observatory (GLOBOCAN) database (https://gco.iarc.fr/today/, accessed on 26 September 2020), United Nations Development Program/Human Development Report Office (http://hdr.undp.org/en, accessed on 26 September 2020) andWorld Health Statistics database (https://www.who.int/gho/publications/world_health_statistics/en/, accessed on 26 September 2020).
